# Novel mechanism of napabucasin, a naturally derived furanonaphthoquinone: apoptosis and autophagy induction in lung cancer cells through direct targeting on Akt/mTOR proteins

**DOI:** 10.1186/s12906-022-03727-6

**Published:** 2022-09-30

**Authors:** Korrakod Petsri, Sunisa Thongsom, Satapat Racha, Supakarn Chamni, Saresa Jindapol, Nantawat Kaekratoke, Hongbin Zou, Pithi Chanvorachote

**Affiliations:** 1 Center of Excellence in Cancer Cell and Molecular Biology, Faculty of Pharmaceutical Sciences, Chulalongkorn University, Bangkok, 10330 Thailand; 2 Department of Pharmacology and Physiology, Faculty of Pharmaceutical Sciences, Chulalongkorn University, Bangkok, 10330 Thailand; 3 Interdisciplinary Program in Pharmacology, Graduate School, Chulalongkorn University, Bangkok, 10330 Thailand; 4 Department of Pharmacognosy and Pharmaceutical Botany, Faculty of Pharmaceutical Sciences, Chulalongkorn University, Bangkok, 10330 Thailand; 5 Natural Products and Nanoparticles Research Unit (NP2), Chulalongkorn University, Bangkok, 10330 Thailand; 6 Department of Materials Science and Engineering, Vidyasirimedhi Institute of Science and Technology (VISTEC), Rayong, 21210 Thailand; 7 College of Pharmaceutical Sciences, Zhejiang University, Hangzhou, 310058 People’s Republic of China

**Keywords:** Napabucasin, Furanonaphthoquinone, Lung cancer, Anti-cancer, Apoptosis, Autophagy, PI3K/Akt/mTOR pathway, Molecular docking analysis

## Abstract

**Background:**

Akt and mTOR are aberrantly activated in cancers and targeting these proteins are interesting for cancer drug discovery. Napabucasin (NB), a phytochemical compound, has been reported as potential anti-cancer agent, however, Akt and mTOR targeting mechanisms remain unclear.

**Method:**

Apoptosis induction was investigated by Hoechst 33342/PI double staining and annexin V/PI staining with flowcytometry. Autophagy was evaluated by monodansylcadaverine staining and Western blot analysis. Binding affinity of NB and essential signaling proteins (PI3K, Akt, and mTOR) was investigated using molecular docking and confirmed by Western blot analysis.

**Result:**

A structure modification from changing methyl moiety of acetyl group of NB to hydroxyl moiety of carboxyl group of NB derivative (napabucasin-acid or NB-acid) greatly affected the compound activities. NB showed more potent anti-cancer activity. NB reduced cell viability with an approximately 20 times lower IC_50_ and inhibited the colony formation capacity much more than NB-acid treated cells. NB induced cell apoptosis, which was accompanied by decrease Bcl‑2 and Mcl-1 and clevage of PARP, while NB-acid show lesser effect on Mcl-1. NB was found to strongly induce autophagy indicated by acidic vesicle staining and the LC3B conversion. Interestingly, computational molecular docking analysis further demonstrated that NB directly bound to Akt and mTOR (complex 1 and 2) proteins at their critical sites indicating that NB targets the upstream regulators of apoptosis and autophagy. The docking results were confirmed by decrease of p-Akt/Akt, p-mTOR/mTOR, and c-Myc a downstream target of Akt protein levels.

**Conclusion:**

Results show for the first time that NB exerts an anti-cancer activity through the direct interaction to Akt and mTOR proteins. The methyl moiety of acetyl group of NB is required for its potent anti-cancer activities. These data encourage further development of NB compounds for Akt and mTOR driven cancers.

**Supplementary Information:**

The online version contains supplementary material available at 10.1186/s12906-022-03727-6.

## Introduction

As the leading cause of cancer-related death reported by the worldwide record, lung cancer acquires an estimated death more than 20% in male and 10% in female of all cancer cases [1, 2]. In spite of advanced therapeutic strategies, the 5-year survival rate is still low according to chemotherapeutic resistance and cancer recurrence [2]. Consequently, novel strategies for cancer treatment are critically required.

Among the several aspects of cancer therapy, targeting on apoptotic cell death is one of the most effective ways because apoptosis evasion is a prominent hallmark for all types of cancer including lung cancer [3, 4]. It has been known that apoptosis program is well-controlled cell death mechanism for homeostasis by the removal of unwanted or harmful cells. Thus, dysregulation of apoptotic pathway results in sustained cell proliferation and enhanced tumor development [3]. To treat cancers, it is necessary to regain the control of cell growth or terminate the uncontrolled cancer cells by restoration of apoptotic signaling pathway.

Not only apoptosis, but targeting on autophagy are also mentioned as mechanisms determining the survival or death of cancer cells. Although autophagy catalytic process enhances cell survival through the recycling of cellular units and bioenergetics and by removing misfolded or aggregated proteins and clearing damaged organelles [5], several evidences also suggest that autophagy is critical for cell death mechanism, especially in the case of inhibition and defection of apoptosis mechanism [6]. Autophagy has been reported to be a mechanism for cell death induction in chemotherapeutic resistant cancer cells [7, 8]. Many studies have found that autophagy and apoptosis play a collaborative role in the treatment of cancer [9]. The induction of autophagy occurs with the induction of apoptosis and does not have an active role in it is defined as autophagy-associated cell death [10]. Certain compounds that induce autophagy-associated cell death have been shown to be beneficial for cancer treatment [11–13].

Apoptosis and autophagy are both regulated by phosphatidyl inositol 3-kinase (PI3K)/Akt/mTOR signaling pathway [14]. Inhibition of key survival proteins such as Akt and mTOR is a promising strategy for cancer treatment since this pathway is the most frequently found dysregulated in cancers including lung cancer. In lung cancer, the alteration of upstream regulators, such as activating the epidermal growth factor receptor (EGFR) can lead to sustained activation of the PI3K/Akt/mTOR cascade [15]. Studies indicated that phosphorylated Akt observed in non-small cell lung cancer (NSCLC) specimens was associated with poor prognosis [16]. The over-activation of the key molecules in this pathway unnecessarily promotes cancer cell survival by cell death inhibition [17]. Once Akt and mTOR activation occur, it mediates the inhibition and activation of several proteins such as pro-apoptotic protein inhibition (Bim, Bax, and Bad), anti-apoptotic protein activation (Bcl-2 and Mcl-1) [18], and pro-autophagic protein inhibition (ULK1 complex) [19] resulting in evasion of apoptosis and autophagy. The key checkpoint for the negative regulation of autophagy is mTOR. Several anti-cancer drugs activate autophagy by inhibiting the PI3K/Akt/mTOR pathway [20]. Taken together, inhibition of this survival pathway can lead to promotion of cell death in cancer cells via apoptosis and autophagy. Many evidences supported this idea. Inhibition of PI3K/Akt/mTOR pathway could drive cell death process of cancer cells and could slow the progress of tumors in vivo [21–23]. Akt and mTOR targeted drugs were developed for cancer treatment [24]. Therefore, the compounds with an ability to induce apoptosis and autophagy by inhibiting PI3K/Akt/mTOR pathway are of interest as good candidates for lung cancer therapy. 

Napabucasin (NB) is a plant-produced furanonaphthoquinone found in Bignoniaceae family and several botanical sources including *Tabebuia cassinoides* [25]*, Millettia versicolor* [26]*, Ailanthus integrifolia* [27]*, Ekmanianthe longiflora* [28], *Newbouldia laevis* [29], and *Handroanthus impetiginosus* [30]. Previous study demonstrated that NB and other compounds isolated from the root of *Ekmanianthe longiflora* showed a potent cytotoxicity among cancer cell lines including colon cancer, lung cancer, and multidrug resistant oral epidermoid carcinoma [28]. Additionally, compounds with the same core structure of NB extracted from *Newbouldia laevis* exhibited moderate antibacterial and herbicidal against gram-positive *Bacillus megaterium* [31]. Recently, NB has been reported as a small molecule inhibitor of STAT3 which has been shown to suppress cancer stemness in several cancer types because STAT3 regulates the expression of proteins involved in cancer stem cell (CSC) self-renewal [32–35]. It has been demonstrated that NB could block spleen and liver metastasis in mouse model of colon cancer and also inhibit signaling pathways such as Nanog, sex-determining region Y-box protein 2 (Sox2), c-Myc, and β-catenin [35]. Phase I-III clinical trials have been undergoing with napabucasin in combination with standard chemotherapies to treat cancers especially cancer in gastrointestinal (GI) tract [36, 37]. However, there are no evidence involved with PI3K/Akt/mTOR pathway inhibition through the direct interaction with key proteins of this cascade in lung cancer. Besides, structure–activity relationships (SARs) of NB is still unclear. Understanding the structure–activity relationships (SARs) is useful for identification of the active moieties which are critical for drug action. Herein, apoptotic and autophagic induction effects together with effects on targeting essential proteins in PI3K/Akt/mTOR pathway on NSCLC cells were investigated in this research. Napabucasin (NB) and napabucasin-acid (NB-acid) which is NB derivative that replaces acetyl group with a carboxyl group were synthesized and evaluated in comparison for the SARs establishment. The obtained information would facilitate the novel aspect of napabucasin as a targeted therapeutic drug for treatment of lung cancer and support further development of NB related compounds along with the design of novel drugs.

## Materials and methods

### Synthesis of napabucasin (NB) and napabucasin-acid (NB-acid)

2-Acetylnaphtho[2,3-*b*]furan-4,9-dione (Napabucasin, NB) and 4,9-Dihydro-4,9-dioxonaphtho[2,3-*b*]furan-2-carboxylic acid (NB-acid) were synthesized according to previously described procedures [38–40]. NB was obtained as a yellow powder at 420 mg, whereas NB-acid was afforded as an orange solid at 160 mg after a purification by silica gel column chromatography. The spectroscopic data of NB and NB-acid was matched with the previous reports [38, 40].

### Reagents and antibodies

Dulbecco’s Modified Eagle’s Medium (DMEM), Roswell Park Memorial Institute (RPMI) 1640 medium, l-glutamine, fetal bovine serum (FBS), antibiotic–antimycotic, trypsin–EDTA, and phosphate-buffered saline (PBS) for cell culture were purchased from Gibco (Grand Island, NY, USA). Dimethyl sulfoxide (DMSO), 3-(4,5-dimethylthiazol-2-yl)-2,5-diphenyltetrazoliumbromide (MTT), Hoechst 33342, propidium iodide (PI), monodansylcadaverine, rapamycin, chloroquine, and bovine serum albumin (BSA) were purchased from Sigma-Aldrich, Co. (St. Louis, MO, USA). RIPA buffer, primary antibodies (Mcl-1 (#94,296), Bcl-2 (#4223), Bax (#5023), PARP (#9532), LC3B (#2775), PI3K (#4292), p-PI3K (#4228), Akt (#9272), p-Akt (#4060), mTOR (#2983), p-mTOR (#5536), c-Myc (#18,583), and β-actin (#4970)), and secondary antibody (anti-rabbit IgG, HRP-linked (#7074)) were purchased from Cell Signaling Technology (Danvers, MA, USA). In addition, Annexin V-FITC/PI apoptosis kit was obtained from ImmunoTools (Gladiolenweg 2, Friesoythe, Germany).

### Preparation of the compound stock solution

The 50 mM stock solutions of NB and NB-acid were prepared by dissolving in dimethyl sulfoxide (DMSO) solution and stored at -20 °C. Then, they were diluted to achieve the final concentrations in culture medium before using for the treatment. The final concentration of DMSO was 0.2% solution, which showed no signs of cytotoxicity.

### Cell lines and culture

Human non-small cell lung cancer (NSCLC) cell lines (H460, H292, H23, and A549), human bronchial epithelial cell line (BEAS-2B), and human endothelial cell line (EA.hy926) were purchased from the American Type Culture Collection (Manassas, VA, USA). H460, H292, and H23 cells were cultured in RPMI, whereas A549, BEAS-2B, and EA.hy926 cells were cultured in DMEM. The both cultured mediums were supplemented with 10% FBS, 100 units/mL of antibiotic–antimycotic, and 2 mM of L-glutamine. The cells were maintained in a humidified incubator at 37 °C with 5% CO_2_.

### Cell viability assay

Cell viability was investigated by MTT assay, which measures cellular metabolic activity to reduce MTT into a formazan product by mitochondria dehydrogenase enzyme. NSCLC, BEAS-2B, and EA.hy926 cells were seeded with a density of 1 × 10^4^ cells/well into 96-well plates for overnight. Several concentrations of NB and NB-acid (0, 1, 5, 10, 25, 50, and 100 µM) were used to treat the cells for 24 h. In the conditions that rapamycin (0.2 µM) and chloroquine (10 µM) were used, they were added to pretreat the cells for 1 h before treatment with NB for 24 h. After the medium was removed, 100 µL of MTT solution (0.5 mg/mL) was added to each well and then incubated for 3 h at 37 °C in dark. Subsequently, MTT solution was removed and 100 µL of DMSO was added to dissolve the formazan crystals of MTT. A microplate reader (Anthros, Durham, NC, USA) was utilized to measure the optical density at 570 nm. The half maximal inhibitory concentration (IC_50_) and the percentage of cell viability were calculated according to the manufacturer’s protocol (7sea Biotech). Some concentrations of NB-acid and NB were selected for the next experiments.

### Colony formation assay

Survival ability and capability of a single cancer cell to proliferate into a colony were examined using colony formation assay. The single cell suspension containing 300 viable NSCLC cells was placed onto 6-well plate for overnight. Several concentrations of NB and NB-acid (0, 1, 5, 10, and 25 µM) were used to treat the cells for 24 h. After that, the medium was removed and the cells were further cultured at 37˚C with 5% CO_2_ for 7 days. 4% paraformaldehyde and 0.05% w/v of crystal violet were used to fix and stain the forming colonies for counting the colony number.

### Nuclear staining assay

The fluorescent detection by Hoechst 33342 and propidium iodide (PI) double staining was used to screen for apoptosis. NSCLC cells were seeded in 96-well plate with a density of 1 × 10^4^ cells/well for overnight and further treated with several concentrations of NB and NB-acid (0, 1, 5, and 10 µM) for 24 h. In the conditions that rapamycin (0.2 µM) and chloroquine (10 µM) were used, they were added to pretreat the cells for 1 h before treatment with NB for 24 h. Then, Hoechst 33342 (10 µg/mL) was added to stain the cells and incubated for 15 min at 37 °C before adding PI (5 µg/mL) and immediately detecting fluorescence by fluorescent microscope (Nikon ECLIPSE Ts2, Tokyo, Japan). The cells presented with nuclear chromatin condensation and DNA fragmentation were counted and calculated to the percentages.

### Annexin V-FITC/PI staining apoptotic assay

The cells undergoing apoptosis process were determined using Annexin V-FITC/PI apoptosis kit (ImmunoTools, Gladiolenweg 2, Friesoythe, Germany). NSCLC cells were seeded in 24-well plate with a density of 5 × 10^4^ cells/well for overnight and then treated with several concentrations of NB and NB-acid (0, 1, 5, 10, and 25 µM) for 24 h. After that, the cells were collected and suspended in 100 µL of annexin V-FITC binding buffer. Then, annexin V-FITC and PI were added and incubated for 15 min at room temperature as recommended in the manufacturer’s protocol (ImmunoTools, Gladiolenweg 2, Friesoythe, Germany). Guava easyCyte flow cytometer (EMD Millipore, Hayward, CA, USA) was used to analyze the cells.

### Western blot analysis

Western blot analysis was used to determine protein expression levels in the cells after treatment. NSCLC cells (H460) were seeded in 6-well plate with a density of 4 × 10^5^ cells/well for overnight and then treated with several concentrations of NB and NB-acid (0, 1, 5, and 10 µM) for 6 and 24 h. After the treatment time, the cells were lysed with radioimmunoprecipitation assay (RIPA) lysis buffer which contained a protease inhibitor cocktail (Roche Diagnostics, Indianapolis, IN, USA), 1% sodium deoxycholate, 1% NP-400, 1% SDS, 150 mM NaCl, and 25 mM Tris–HCl pH 7.6 for 30 min at 4 °C to extract the proteins. The cell lysates were collected and protein concentrations were measured by a BCA protein assay kit (Pierce Biotechnology, Rockford, IL, USA). Equal amount of denatured proteins (70 µg) was loaded and separated by SDS–polyacrylamide gel electrophoresis and then transferred to 0.2 µm polyvinylidene difluoride (PVDF) membranes (Bio-Rad Laboratories, Hercules, CA, USA). 5% skim milk in TBST (Tris-buffer saline containing 125 mM NaCl, 25 mM Tris–HCl pH 7.5, and 0.1% tween 20) was used to block the non-specific binding on the blots for 1 h at room temperature. After that, the membranes were incubated with specific primary antibodies (Bcl-2, Mcl-1, Bax, PARP, LC3B, PI3K, p-PI3K, Akt, p-Akt, mTOR, p-mTOR, c-Myc, and β-actin at 4 °C for overnight. After incubation, the membranes were washed three times with TBST and further incubated with secondary antibody (anti-rabbit IgG, HRP-linked) for 2 h at room temperature. The enhanced chemiluminescent detection system (SupersignalWest Pico, Pierce, Rockford, IL, USA) was utilized to detect the immunoreactive proteins which further exposed to X-ray film. The protein band intensity was examined using ImageJ software (version 1.52, National Institutes of Health, Bethesda, MD, USA). Densitometric values were calculated relative to β-actin.

### Monodansylcadaverine staining

Monodansylcadaverine (MDC), a fluorescent compound, was used as a probe to detect autophagic vacuoles in cultured cells. NSCLC cells (H460) were seeded in 96-well plate with a density of 1 × 10^4^ cells/well for overnight and treated with several concentrations of NB (0, 1, 5, and 10 µM) for 24 h. Subsequently, the cells were stained with MDC (50 µM) and incubated for 30 min at 37 °C before immediately detecting fluorescence by fluorescent microscope (Nikon ECLIPSE Ts2, Tokyo, Japan).

### Molecular docking analysis

The 3D structure of NB was obtained from the PubChem compound database (CID: 10,331,844) [41], whereas the protein structures of Akt1 (PDB: 3CQW) [42], the pleckstrin homology domain of Akt-1 (PDB: 1UNQ) [43], mTORC1 (PDB: 6BCU) [44], and mTORC2 (PDB: 6ZWM) [45] were obtained from the Research Collaboratory for Structural Bioinformatics Protein Data Bank [46]. The UCSF ChimeraX [47] was used to generate all protein structures for docking and Autodock Vina [48] was used to explore the molecular interaction of ligands and proteins. The exhaustiveness parameter was left at its default value and the grid size was set to 20 20 20 with a spacing of 1. The binding site was created using the ligand's core from the PDB structure. Additionally, 3D binding interactions were shown using the UCSF ChimeraX.

### Statistical analysis

The data from three independent replicated experiments (*n* = 3) was demonstrated as the mean ± standard deviation. Statistical differences were determined by an analysis of variance (ANOVA). A significance level of *p* < 0.05 was considered as statistical significance. GraphPad prism (GraphPad Software, San Diego, CA, USA) and SPSS software program version 16 (SPSS Inc., Chicago, IL, USA) were used to generate graphs and analyze the data.

## Results

### Synthesis of NB and NB-acid

**Fig. 1:**
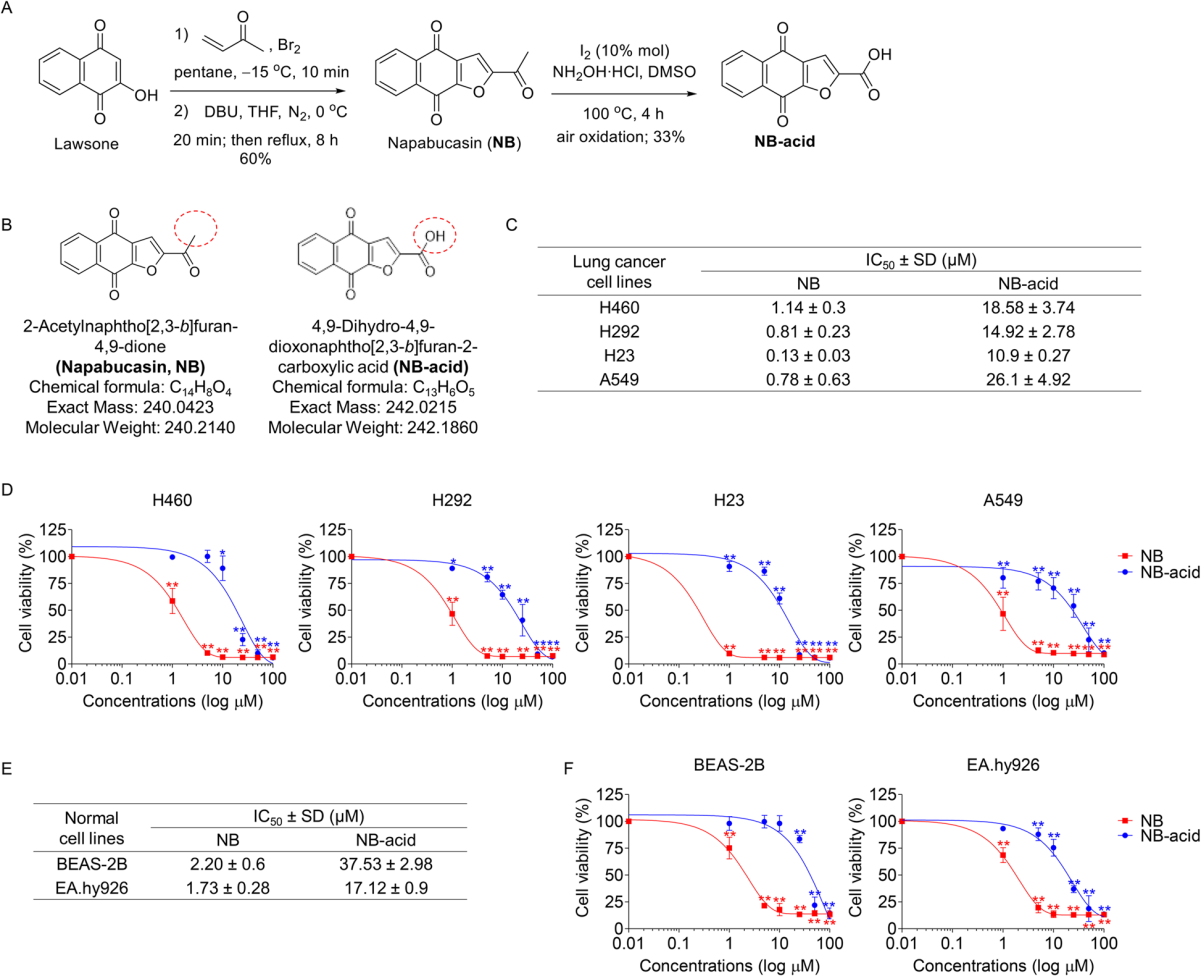
Synthesis of NB and NB-acid and their effects on cell viability in NSCLC cells (H460, H292, H23, and A549), human bronchial epithelial cell line (BEAS-2B), and human endothelial cell line (EA.hy926). (**A**) Synthesis of NB and NB-acid (**B**) Structures of NB and NB-acid (**C**-**F**) The cells were seeded and treated with 0–100 µM of NB-acid and NB for 24 h. Then, the MTT assay was performed to examine the percentages of cell viability. IC_50_ of each compounds was calculated compared to the untreated control. Data represent the mean ± SD (*n* = 3) (* 0.01 ≤ *p* < 0.05, ** *p* < 0.01, compared with the untreated control)

Napabucasin (NB) was obtained from herbal extraction or synthesis (Fig. 1A). Based on synthetic approach, lapacol was used as a precurrsor for the one-pot michael-*O*-alkylation to afford NB [38]. Subsequently, transformation of acetyl group of NB to caboxyl group by iodine-catalyzed oxidative C–C bond cleavage yielded NB-acid [39]. The structures of NB and NB-acid were presented in Fig. 1B.

### Cytotoxicity and inhibition of colony forming capacity effects of NB and NB-acid

Firstly, to investigate and confirm the anti-cancer potential of NB and NB-acid on lung cancer cells, the cytotoxic profile of NB and NB-acid in several lines of NSCLC cells including H460, H23, H292, and A549 was determined. Moreover, normal somatic cells including BEAS-2B and EA.hy926 cells were used for comparing the toxicity between normal cells and cancer cells. After treatment the cells with NB and NB-acid for 24 h, MTT assay was used to evaluate the percentage of cell viability. The results demonstrated that NB was shown more potent effects than NB-acid. The significant diminution of cell viability in NSCLC cells was presented at the very low concentration (1 μM) with the IC_50_ at 1.14 ± 0.3, 0.81 ± 0.23, 0.13 ± 0.03, and 0.78 ± 0.63 μM in H460, H292, H23, and A549 cells, respectively (Fig. 1C and D). Meanwhile, NB-acid could significantly reduce cell viability in NSCLC cells (H460, H292, H23, and A549) at the low concentrations (1–10 μM) compared to untreated control with the IC_50_ as following; 18.58 ± 3.74, 14.92 ± 2.78, 10.9 ± 0.27, and 26.1 ± 4.92 μM in H460, H292, H23, and A549 cells, respectively (Fig. 1C and D). In human bronchial epithelial cells (BEAS-2B), both compounds showed the 2–3 folds higher IC_50_ than NSCLC cells indicating the selective cytotoxicity (Fig. 1E and F). However, in human endothelial cells (EA.hy926), only NB had the 2 folds higher IC_50_ than NSCLC cells, whereas the IC_50_ of NB-acid was equally to NSCLC cells (Fig. 1E) indicating that NB might be more selectivity than NB-acid in cancer cells.

**Fig. 2:**
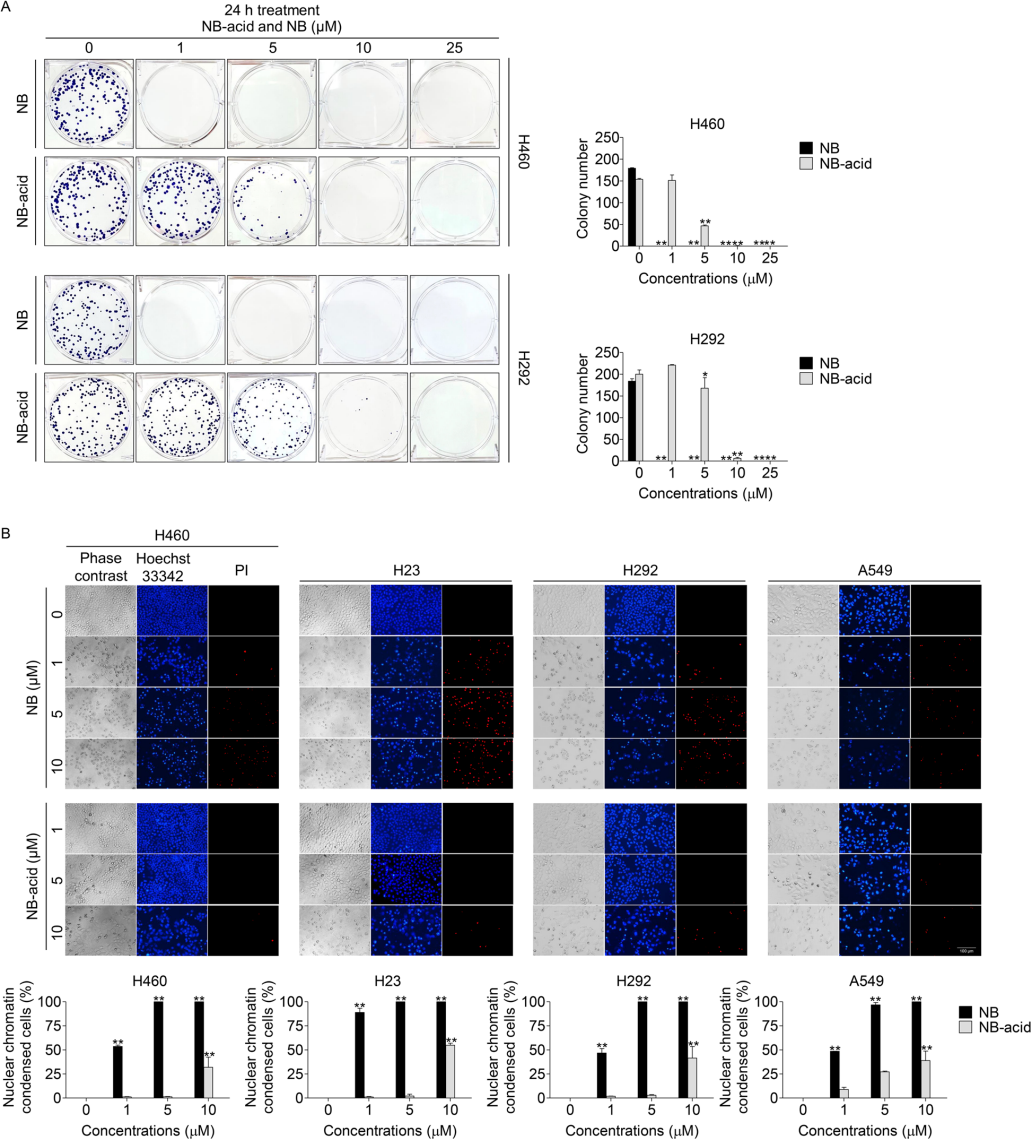
Effects of NB and NB-acid on the inhibition of colony formation and apoptotic induction in NSCLC cells (H460, H292, H23, and A549) (**A**) NSCLC cells (H460 and H292) were seeded and treated with 0–25 µM of NB-acid and NB for 24 h. After that, they were further cultured for 7 days and stained with crystal violet for counting the colony number. (**B**) The cells were seeded and treated with 0–25 µM of NB and NB-acid for 24 h. Then, Hoechst 33,342 and PI were added to stain the cell nucleuses. Images were captured using a fluorescence microscope and the percentages of nuclear condensed and propidium iodide (PI)-positive cells were calculated. Data represent the mean ± SD (*n* = 3) (* 0.01 ≤ *p* < 0.05, ** *p* < 0.01, compared with the untreated control)

Colony formation assay is a cell survival assay based on the principle of the ability of a single cancer cell to grow into a colony. This method can be used to determine the effectiveness of cytotoxic agents [49]. NSCLC cells (H460 and H292) after the treatment with NB and NB-acid (1–25 μM) for 24 h were subjected to colony formation assay for 7 days. The crystal violet-staining colony represented the suppression on colony regeneration was significantly decreased in both compounds compared to untreated control (Fig. 2A). Surprisingly, the absence of colony number was found in every concentration of NB, whereas the forming colony was hardly detected in the treatment at 10 μM of NB-acid. Overall, these results suggested the promising effects of NB and NB-acid as an alternative approach in anti-cancer therapy.

### Apoptosis induction and apoptotic related protein alteration effects of NB and NB-acid

Apoptosis is considered as an important component of various processes since it contributes to elimination of unnecessary and unwanted cells to maintain the balance between cell survival and cell death. It is characterized by morphological changes including nuclei shrinkage, nuclear chromatin condensation, cytoplasmic shrinkage, dilated endoplasmic reticulum, and membrane blebbing. Anti-cancer drugs have the major mechanism based on targeting various aspects of apoptotic related process [3]. Therefore, Hoechst 33342 and PI double staining was used to screen the mode of cell death by monitoring the nucleus morphology of apoptotic cells as well as their membrane integrity. After treatment with NB and NB-acid (1–10 μM) for 24 h, NSCLC cells were co-stained with Hoechst 33342 and PI. Bright blue fluorescence of Hoechst 33342 was clearly observed in the representation of condensed and fragmented chromatin in early stage of apoptosis, whereas a red fluorescence of PI indicated late stage of apoptotic or necrotic cells. The result revealed that NB and NB-acid significantly caused an increase of nuclear chromatin condensed cells at 1–10 µM of NB and 10 µM of NB-acid in NSCLC cells (Fig. 2B). In according to the previous experiment that NB had the potent cytotoxicity effect, NB had also cause more dead cells than NB-acid at the same concentrations (1–10 μM) observing from the red fluorescence of PI indicating the late stage of cell death.

**Fig. 3:**
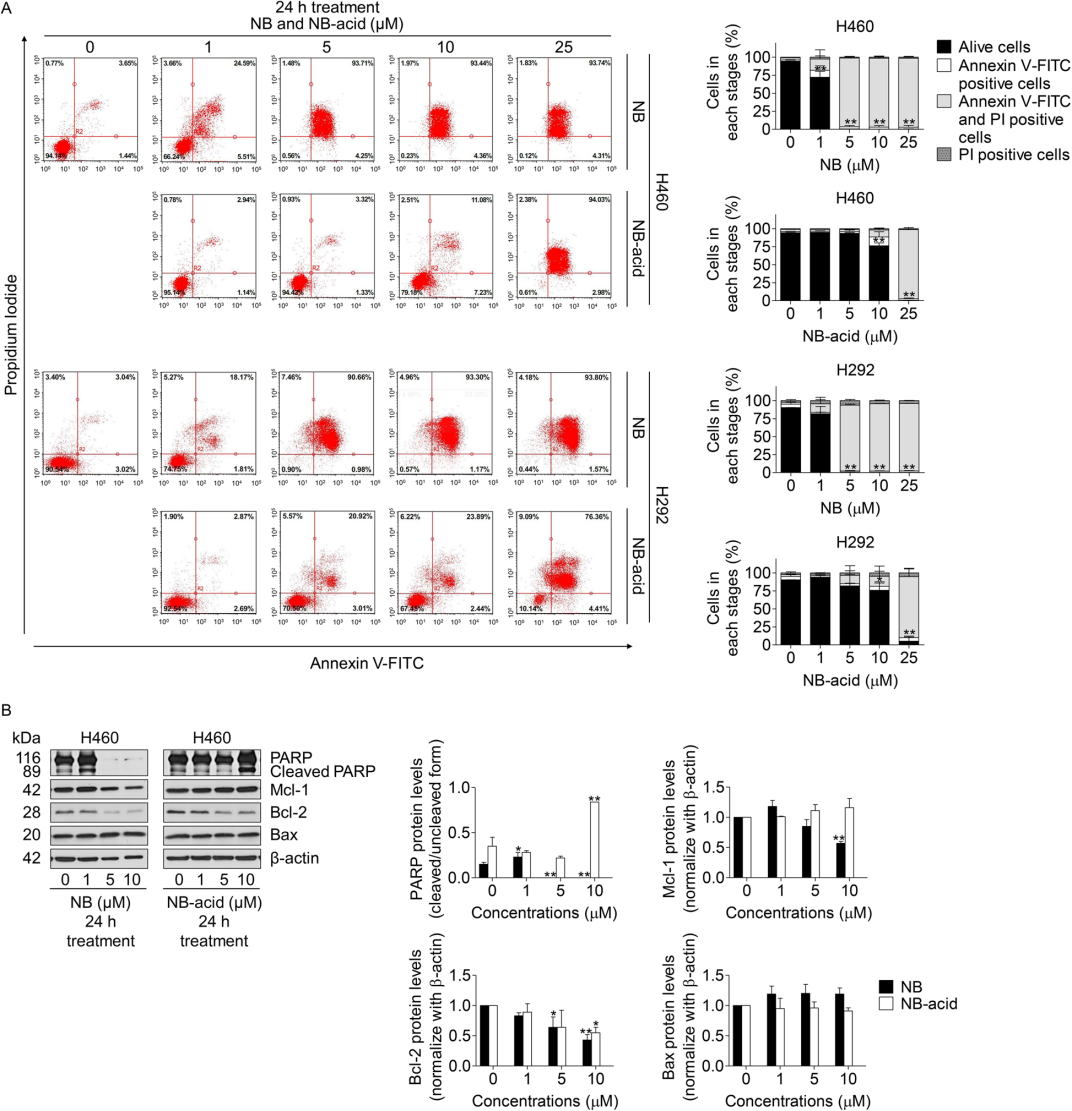
Effects of NB and NB-acid on apoptotic cell death including the alteration of apoptotic related proteins in NSCLC cells (H460 and H292). (**A**) NSCLC cells (H460 and H292) were seeded and treated with 0–25 µM of NB and NB-acid for 24 h. Apoptotic cells were determined using annexin V-FITC/PI staining with flow cytometry. Percentages of cells at each stage were calculated. (**B**) NSCLC cells (H460) were seeded and treated with 0–10 µM of NB and NB-acid for 24 h. Western blot analysis was performed to detect the PARP, cleaved PARP, Mcl-1, Bcl-2, and Bax protein levels. The blots were reprobed with β-actin to confirm an equal loading of each sample. Densitometry was used to calculate the protein expression levels and the values of protein levels were presented as the fold changes relative to uncleaved form of the protein or β-actin. The uncropped blotting bands were presented in Additional file 1. Data represent the mean ± SD (*n* = 3) (* 0.01 ≤ *p* < 0.05, ** *p* < 0.01, compared with the untreated control)

To confirm the apoptotic cell death response to NB and NB-acid treatment, flow cytometric analysis using annexin V/FIT-C and PI staining was utilized. The results were co-related with Hoechst 33342 and PI double staining. NB was found to significantly induce cell death at the concentration of 1 μM, whereas NB-acid caused apoptotic cell death since 10 μM observing from annexin-V/FITC and PI positive cells (Fig. 3A). After that, Western blot analysis was used to investigate the apoptotic-related proteins such as PARP, Mcl-1, Bcl-2, and Bax. The result in Fig. 3B showed an increase of cleaved PARP in response to NB (1 μM) and NB-acid (10 μM) treatment compared to control. However, PARP protein levels in 5–10 μM of NB was diminished. Anti-apoptotic Bcl-2 protein levels in both compounds were found to decrease significantly. On the other hand, anti-apoptotic Mcl-1 protein levels were reduced only in NB treatment condition and pro-apoptotic protein Bax protein levels were unchanged in both compounds. Because of the more effective anti-cancer activities of NB, only NB was chosen to continue on the next experiments.

### Autophagy induction and autophagic related protein alteration effects of NB

**Fig. 4:**
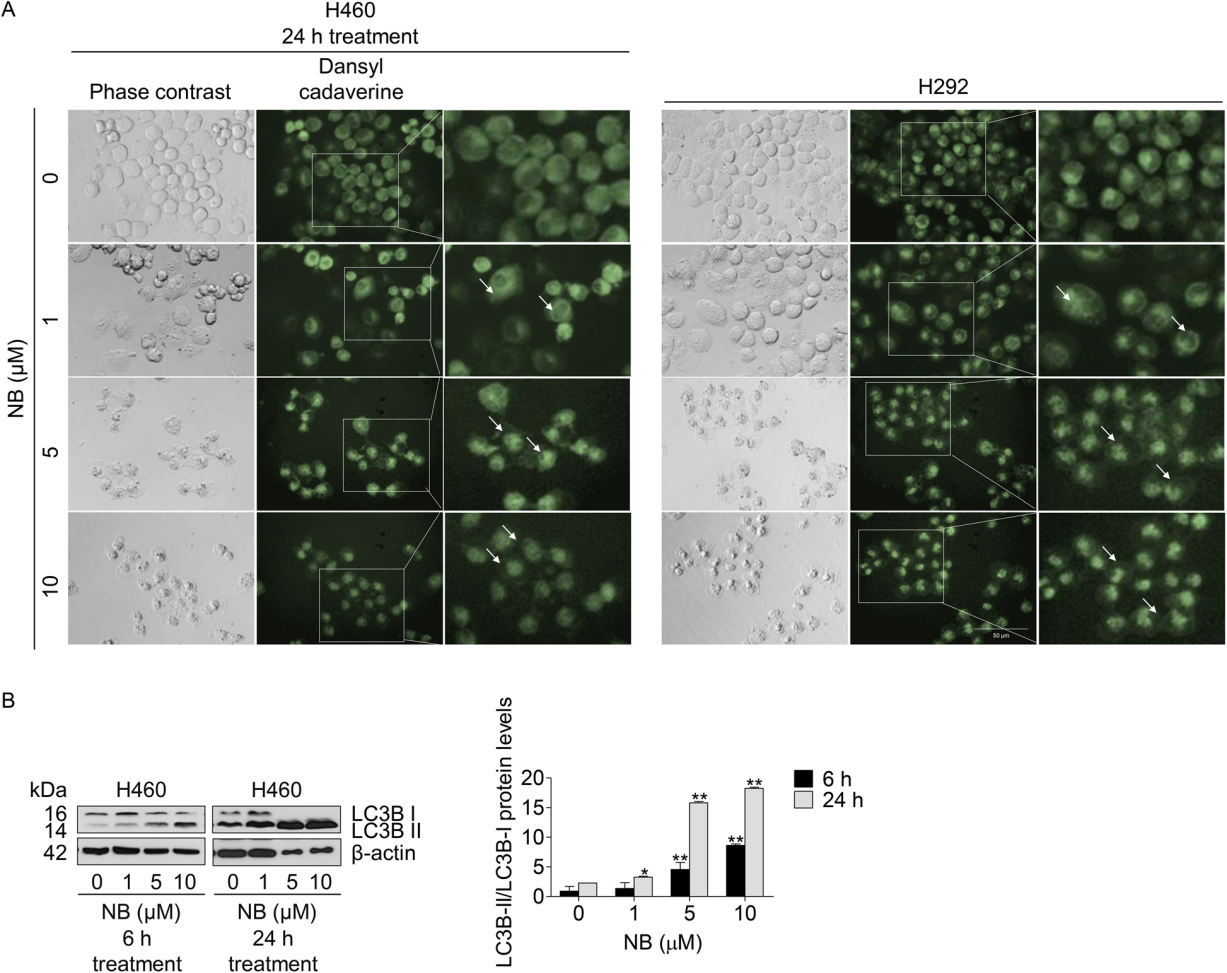
Effects of NB on autophagy including the alteration of autophagic related proteins in NSCLC cells (H460). (**A**) NSCLC cells (H460) were treated with 0–10 µM of NB for 24 h. MDC was added to stained autophagic vacuoles in cultured cells. Images were captured using a fluorescence microscope. (**B**) H460 cells were treated with 0–10 µM of NB for 6 and 24 h. Western blot analysis was performed to detect the LC3B protein levels. The blots were reprobed with β-actin to confirm an equal loading of each sample. The uncropped blotting bands were presented in Additional file 2. Densitometry was used to calculate the protein expression levels and the values of protein levels were presented as the fold changes relative to inactive form of the proteins. Data represent the mean ± SD (*n* = 3) (* 0.01 ≤ *p* < 0.05, ** *p* < 0.01, compared with the untreated control)

Autophagy is an important catabolic process which has a function to digest cellular contents within lysosomes. Autophagy-associated cell death in cancer cells has been proposed as a possible mechanism for achieving cancer elimination. Many anti-cancer drugs affect autophagy by increasing the formation of autophagosomes [50]. MDC was applied to detect the autophagic vacuoles in NSCLC cells. After the treatment with NB, autolysosomes of NSCLC cells were stained with MDC. The results indicated the bright green fluorescence of autophagic vacuoles observed in the NB treated cells compared with those of the untreated control cells (Fig. 4A).

To confirm the autophagy induction of NB, an expression of autophagy related protein marker, LC3B conversion (LC3B I to LC3B II), was determined by Western blot analysis. The results showed an increase of LC3B II that could be clearly observed in concentration- and time-dependent manner after NB treatment (Fig. 4B) confirming that the cells were undergone anautophagy process.

### Molecular docking simulation of the NB interactions with the essential proteins in PI3K/Akt/mTOR pathway

Phosphatidyl inositol 3-kinase (PI3K)/Akt/mTOR pathway has been reported as the main survival pathway which regulates multiple cellular processes including apoptosis and autophagy. The activity of the PI3K/Akt/mTOR pathway can suppress the autophagic and apoptotic process which commonly related to pro-survival effects [14, 51]. Importantly, the over-activation of essential molecules in the pathway unnecessarily promotes cancer cell survival by autophagy and apoptosis inhibition [17]. Inhibition of this survival pathway can lead to promotion of autophagy and apoptosis in cancer cells [52, 53]. From the previous results that NB could induce in both apoptosis and autophagy, the molecular docking would be further performed to screen whether this compound had a capability to bind and inhibit essentials proteins in PI3K/Akt/mTOR pathway or not.

**Fig. 5:**
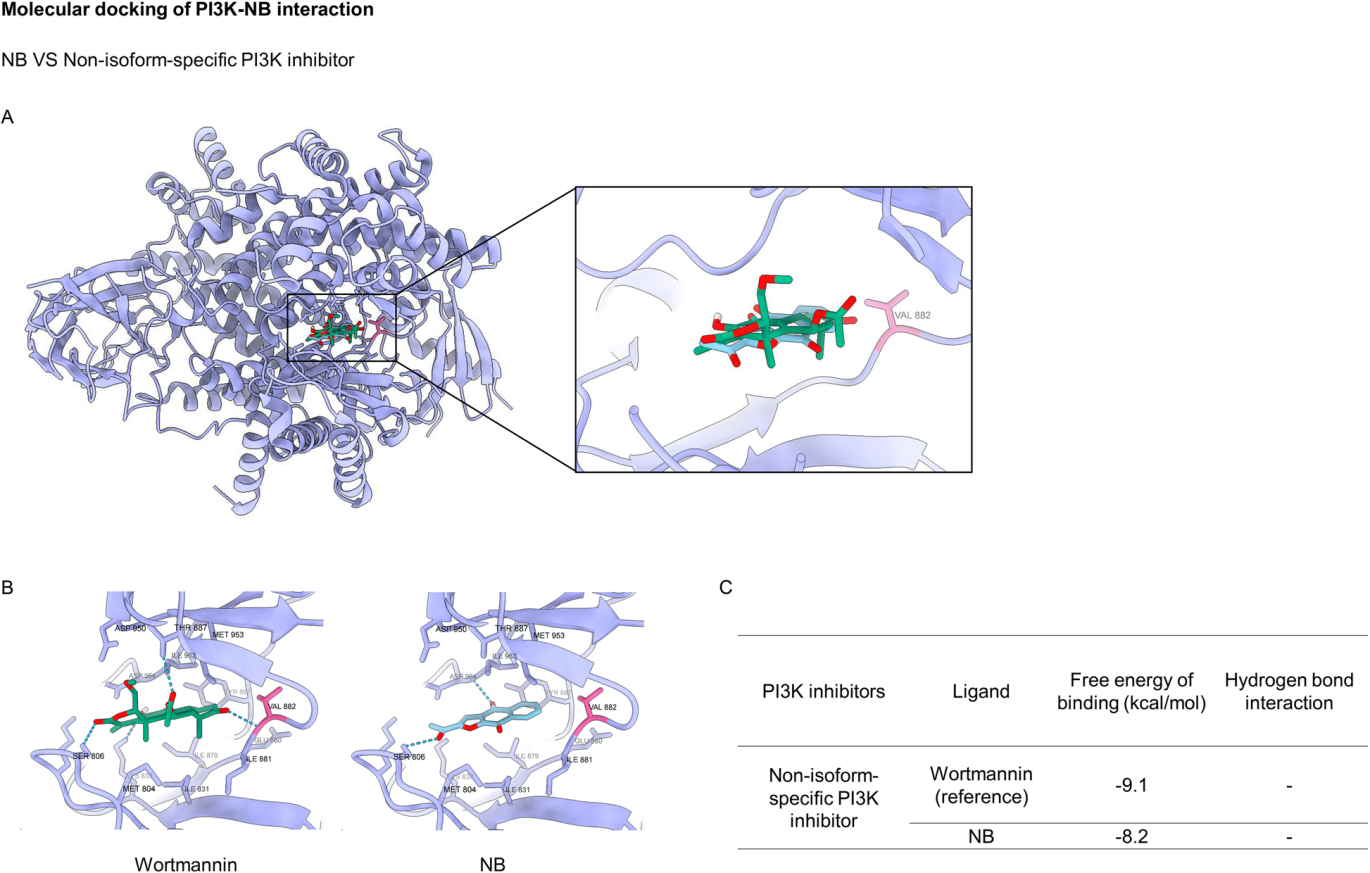
Molecular docking of NB and PI3K compared to non-isoform-specific PI3K inhibitor (wortmannin). (**A**) The 3D binding site of NB and wortmannin. (**B**) PI3K in complexed with wortmannin and PI3K in complexed with NB. The blue dashed lines denoted hydrogen-bonding interaction. Val882 was colored in pink. (**C**) Docking energy of NB and PI3K compared to wortmannin

PI3K, a key molecule in the initiation of signal transduction pathways, was applied as the first target for molecular docking. Wortmannin, a PI3K inhibitor, was employed as a reference molecule in the docking method. The docking results of NB and wortmannin with the PI3K was provided in Fig. 5A-C. Wortmannin had the strongest affinity for the PI3K active site (-9.1 kcal/mol). NB, on the other hand, had the lowest binding affinities (-8.2 kcal/mol) (Fig. 5C) and did not create a hydrogen bond with the Val residue (Fig. 5B). Our findings demonstrated that NB did not bind to PI3K in a significant way.

**Fig. 6:**
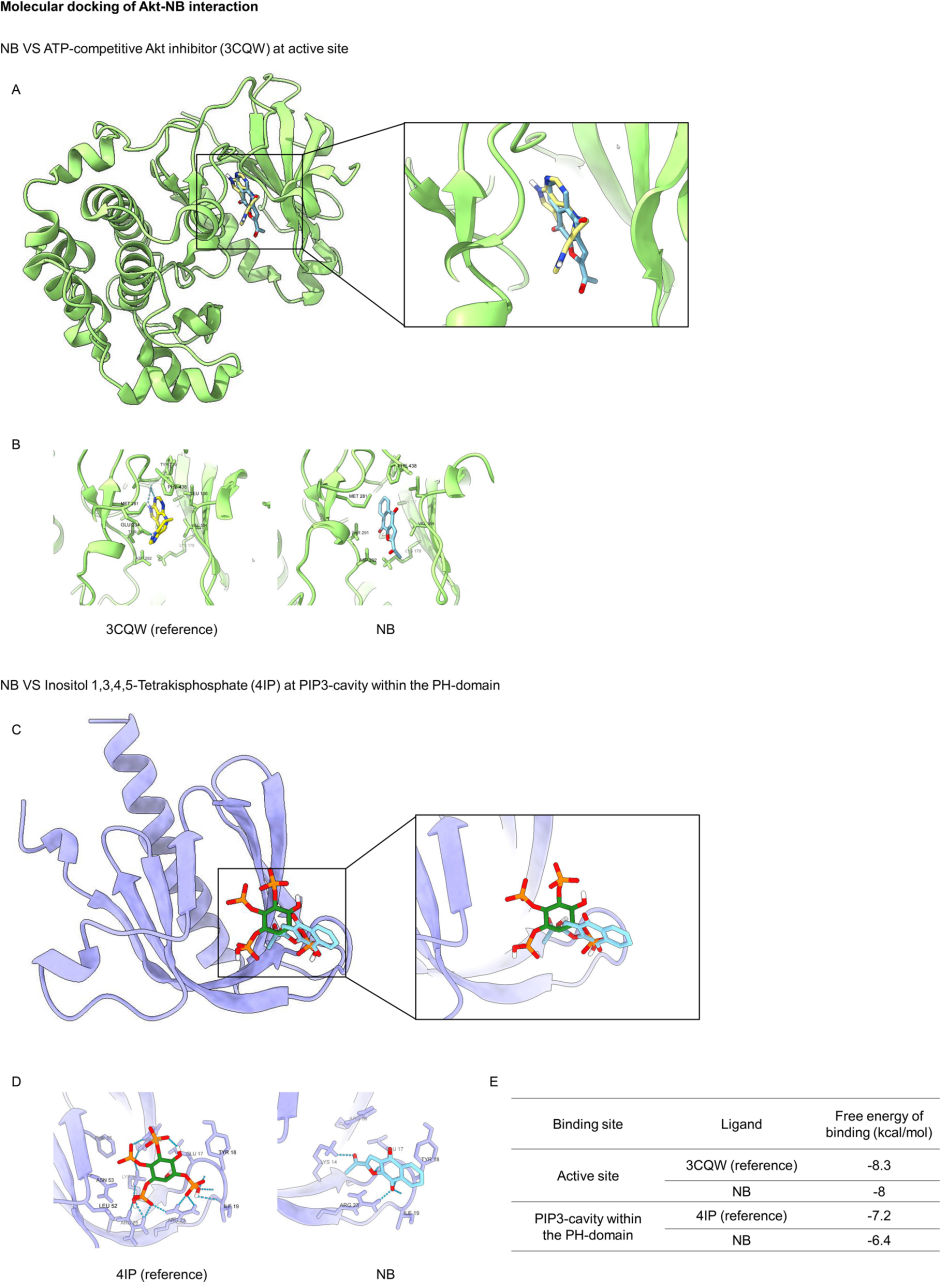
Molecular docking of NB and Akt compared to reference compound. (**A**) The 3D binding site of NB and reference compound at the active site. (**B**) Akt in complexed with reference compound and Akt in complexed with NB at the active site. The blue dashed lines denoted hydrogen-bonding interaction. (**C**) The 3D binding site of NB and 4IP at the PIP3-cavity within the PH-domain. (**D**) PH domain of Akt in complexed with 4IP and PH domain of Akt in complexed with NB. The blue dashed lines denoted hydrogen-bonding interaction. (**E**) Docking energy of NB and Akt compared to references

Akt, a next downstream target of PI3K which usually are a valuable therapeutic target, was further applied. Two mains binding sites of Akt were used as the target for molecular docking; active (ATP binding) site and PIP3-cavity within the PH-domain. The use of ATP-competitive compounds to target the active site of Akt1 is rather widespread [54]. Based on the molecular docking study, the binding affinities of NB and co-crystal ligand CQW were reported in Fig. 6A-B and E. NB was found to interact with Val164, Ala177, Lys179, Met218, Thr291, Asp292, and Phe438 at the ATP-binding site of Akt with a binding affinity of -8.0 kcal/mol, which was similar to the binding site of the reference compound. The result provided that NB might able to interact with Akt at the active site. For the N-terminal pleckstrin homology (PH) domain of Akt, this domain normally binds with equal affinity with the second messengers PtdIns (3,4,5) P3 and PtdIns (3,4) P2, which are produced by insulin and growth factor-mediated phosphoinositide 3-kinase activation (PI3K) [43]. The docking result of NB with the PH domain of Akt was provided in Fig. 6C-E. NB formed one hydrophobic interaction with Arg86, and it was illustrated that the four residues Lys14, Glu17, Tyr18, and Arg23 forming hydrogen bonds in the PH domain played a significant role in the interaction between NB and Akt. The result provided that NB has a potential role in interacting with PH domain of Akt.

**Fig. 7:**
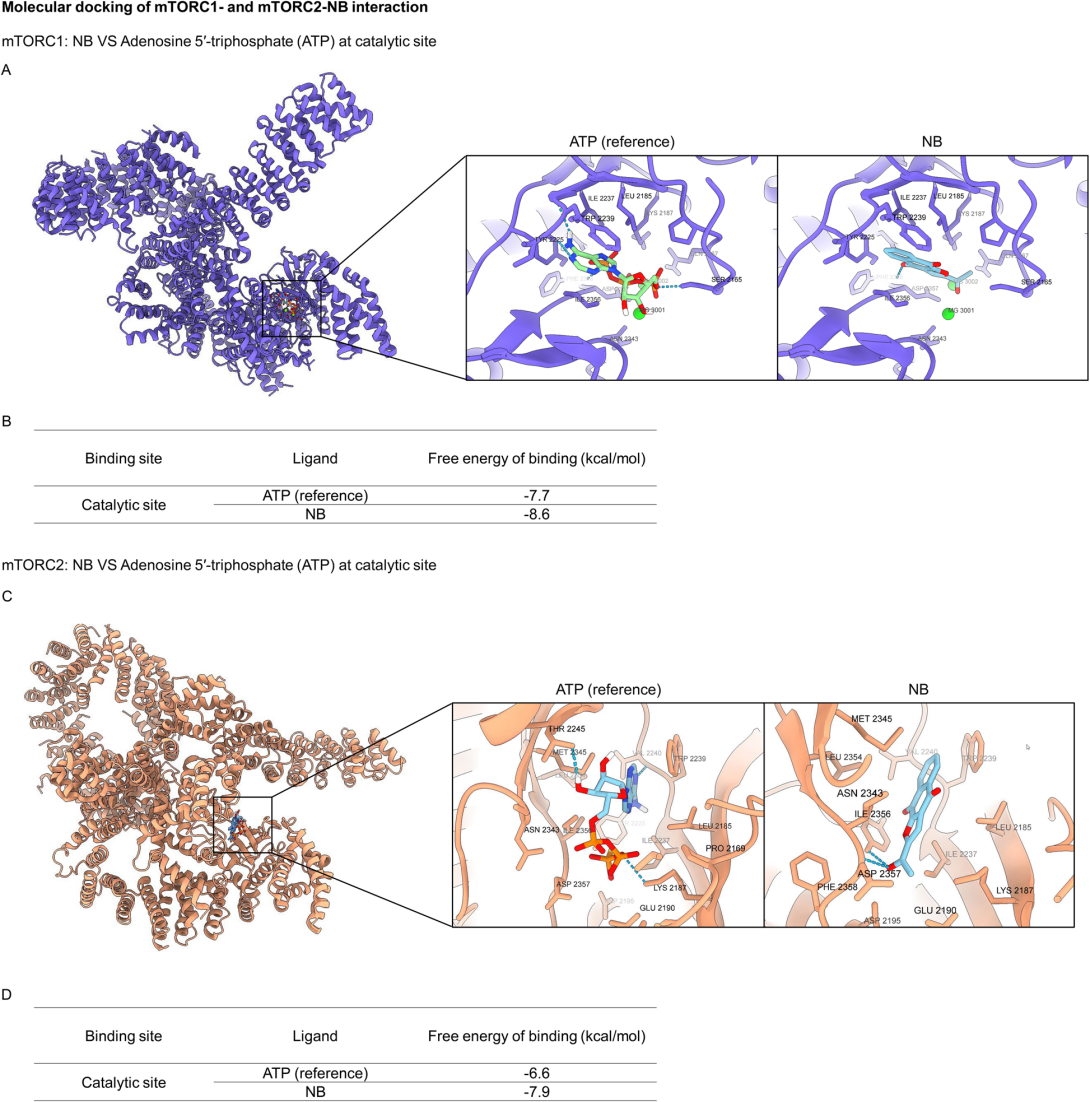
Molecular docking of NB and mTORC1 and mTORC2 compared to reference compound. (**A**) The 3D binding site of NB and ATP in complex with mTORC1 at the catalytic site. ATP-mTORC1 complex and NB-mTORC1 complex. (**B**) Docking energy of NB and mTORC1 compared to reference compound. (**C**) The 3D binding site of NB and ATP in complex with mTORC2 at the catalytic site. ATP-mTORC2 complex and NB-mTORC2 complex. The blue dashed lines denoted hydrogen-bonding interaction. (**D**) Docking energy of NB and mTORC2 compared to reference compound

mTOR has been identified as a druggable target for anti-cancer treatments because it is hyperactivated in response to oncogenic signals and metabolic changes. mTOR is divided into two multi-protein complexes, mTORC1 and mTORC2, which control different molecular processes. mTORC1 which is activated by Akt involves in various cellular outcomes, such as cell cycle progression, proliferation, survival, and apoptosis [55]. Moreover, mTORC1 is found to block autophagy by direct inhibition of the early process and control of the lysosomal degradative capacity of the cells [56]. Therefore, inhibition of mTORC1 would lead to both apoptosis and autophagy. For mTORC2, this complex can cause Akt phosphorylation and influence cytoskeleton protein activities [55]. It has been found that phosphorylation of Akt by mTORC2 at Thr-450 is contributed to Akt stabilization and phosphorylation of Akt by mTORC2 at Ser-473 is essential for Akt activation [57]. Consequently, mTORC2 inhibition would result in Akt inactivation and degradation [58]. The discovery of functional small molecule inhibitors that can compete with ATP for the catalytic target of mTOR to inhibit mTORC1 and mTORC2 kinase is one of the main goals of therapeutic development [55]. The docking results of NB with the catalytic subunit of mTORC1 and mTORC2 were provided in Fig. 7. NB showed the highest binding affinity to both mTORC1 (-8.6 kcal/mol) and mTORC2 (-7.9 kcal/mol) (Fig. 7B and D). For NB-mTORC1 complex, NB contributed to the hydrophobic interactions with Ser2165, Gln2167, Leu2185, Lys2187, Tyr2225, Ile2237, Trp2239, Asn2343, Ile2356, Asp2357, and formed hydrogen bonds with Phe2358 (Fig. 7A). For NB-mTORC2 complex, NB contributed to the hydrophobic interactions with Leu2185, Lys2187, Glu2190, Asp2195, Ile2237, Trp2239, Val2240, Asn2343, Met2345, Leu2354, Phe2358, and formed hydrogen bonds with Ile2356 and Asp2357 (Fig. 7C). The results signified that NB might interrupt the interaction of the ATP complex for the catalytic target of mTOR.

### Akt and mTOR targeting effects of NB mediate apoptosis and autophagy

**Fig. 8:**
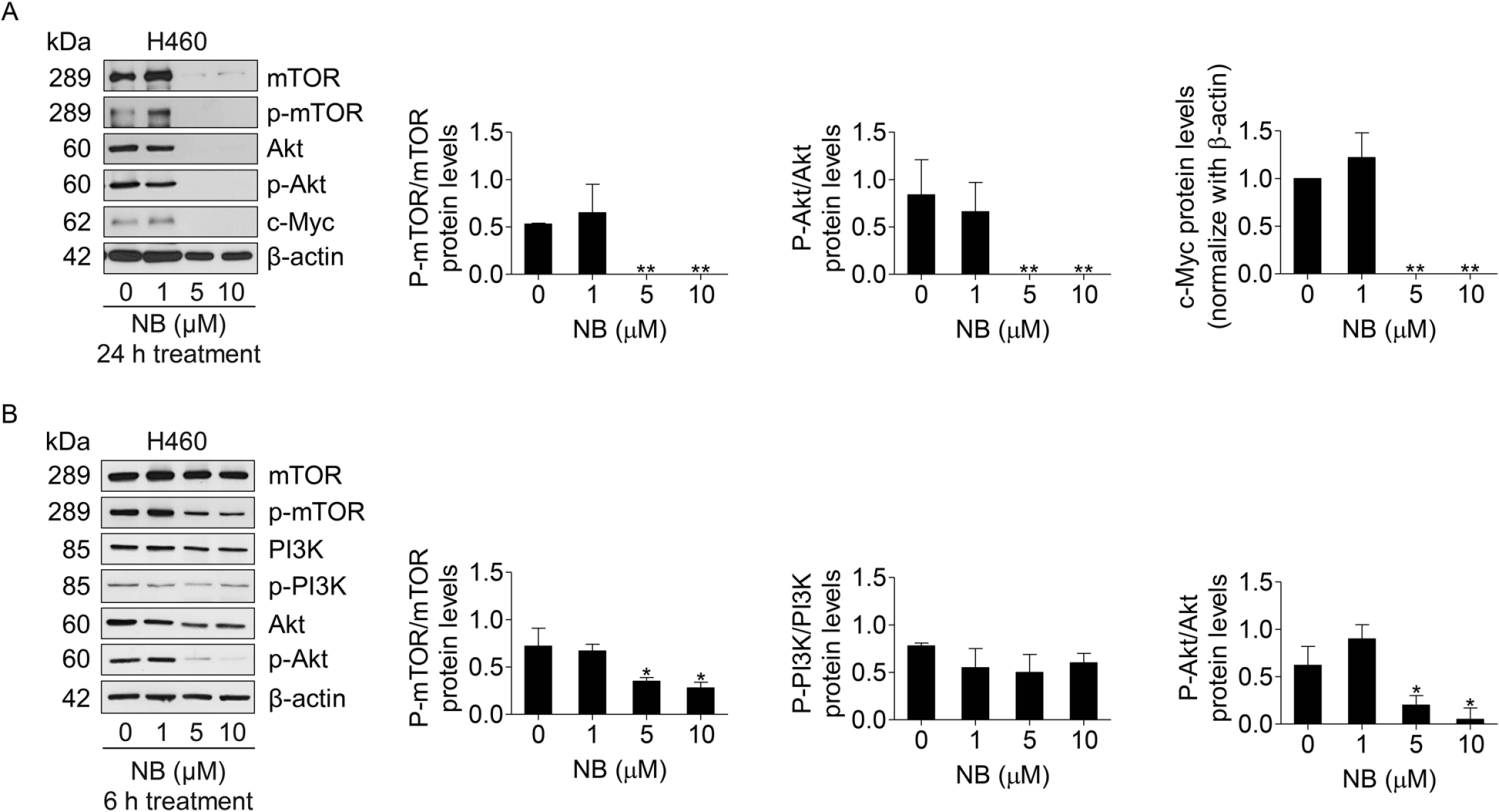
Effects of NB on protein levels of PI3K/Akt/mTOR pathway in NSCLC cells (H460). (**A** and **B**) NSCLC cells (H460) were treated with 0–10 µM of NB for 6 and 24 h. Western blot analysis was performed to detect the mTOR, p-mTOR, PI3K, p-PI3K, Akt, p-Akt, and c-Myc protein levels. The blots were reprobed with β-actin to confirm an equal loading of each sample. Densitometry was used to calculate the protein expression levels and the values of protein levels were presented as the fold changes relative to inactive form of the proteins. The uncropped blotting bands were presented in Additional file 3 and Additional file 4. Data represent the mean ± SD (*n* = 3) (* 0.01 ≤ *p* < 0.05, ** *p* < 0.01, compared with the untreated control)

Having shown the NB ability to bind and inhibit both Akt and mTOR (complex 1 and 2), Western blot analysis was applied to confirm the previous docking results. After treatment with NB for 24 h, Akt, mTOR, and their phosphorylated or active form were detected. Additionally, c-Myc, a downstream survival protein of Akt signaling was also evaluated. The results showed an absence of all survival proteins at 5–10 μM of NB (Fig. 8A). Thus, 6 h treatment was further performed to confirm an early signaling. PI3K and its phosphorylated form which are an initiator of signal transduction pathways were also examined to confirm that NB had no effects on these proteins. The results revealed a significantly decrease of p-Akt/Akt and p-mTOR/mTOR protein expression levels in NSCLC cells at 5–10 μM of NB after 6 h treatment compared to untreated control, whereas no effects of NB on p-PI3K/PI3K was found (Fig. 8B). These results were co-related with the molecular docking that NB induced apoptosis and autophagy through inactivation of Akt and mTOR.

**Fig. 9:**
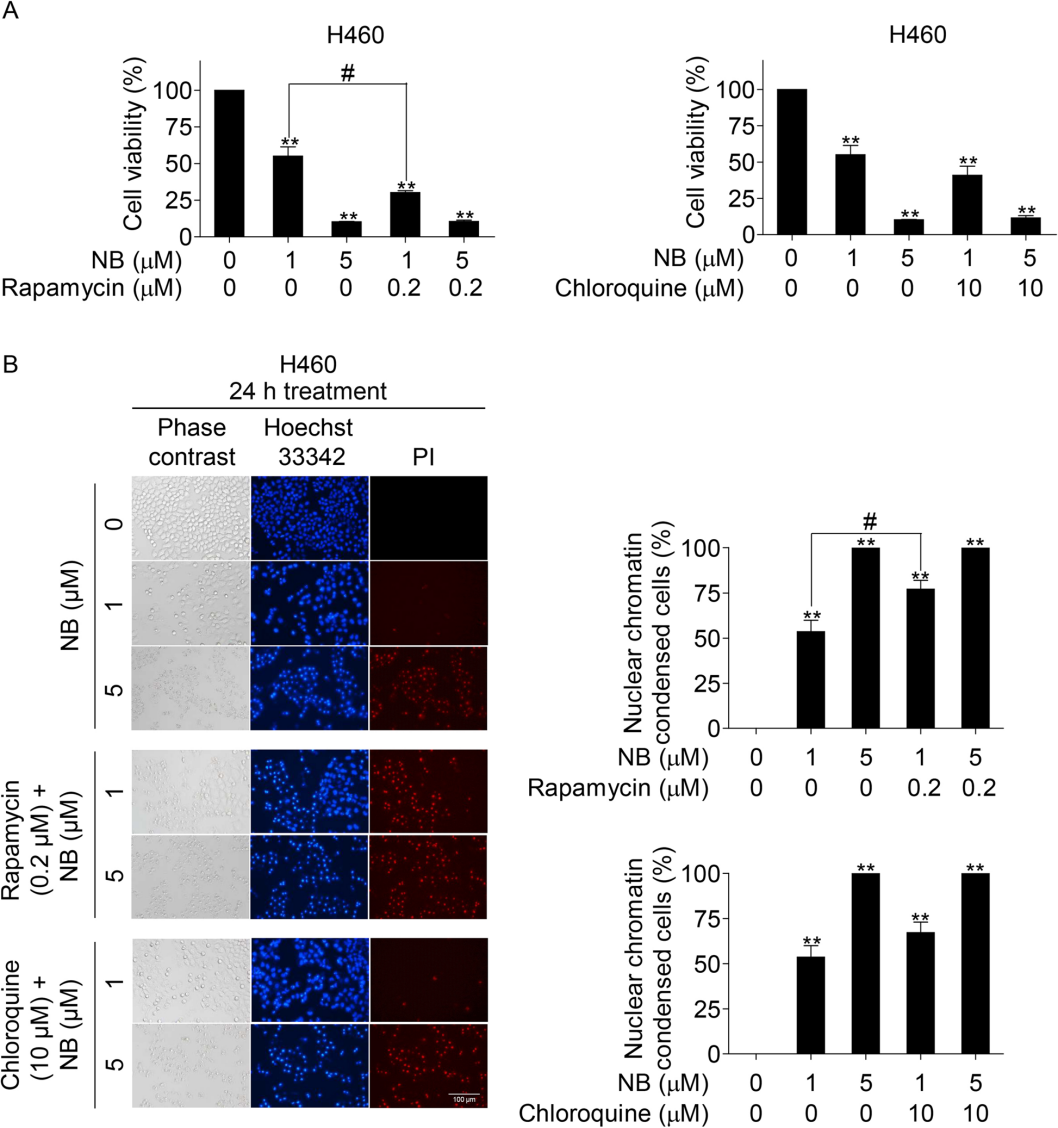
Effects of NB on cell viability and cell death induction in NSCLC cells (H460) in the present of rapamycin and chloroquine (**A** and **B**) H460 cells were seeded and pretreated with rapamycin (0.2 µM) and chloroquine (10 µM) for 1 h before treatment with 0–25 µM of NB for 24 h NB. Then, MTT assay and Hoechst 33,342 and PI staining were performed. Data represent the mean ± SD (*n* = 3) (* 0.01 ≤ *p* < 0.05, ** *p* < 0.01, compared with the untreated control, # 0.01 ≤ *p* < 0.05, compared with NB treatment alone)

To confirm the effects of NB on mTOR, rapamycin (allosteric mTORC1 inhibitor) were utilized (Fig. 9A, B). Rapamycin was found to enhance the cytotoxic effects indicating by the significant increase of cytotoxicity and nuclear chromatin condensed cells. Moreover, chloroquine, an autophagy inhibitor which inhibits autophagic flux by decreasing autophagosome-lysosome fusion, was used. The results demonstrated that treatment with chloroquine in combination with NB did not alter the cytotoxicity effects of NB, indicating by cell viability and nuclear chromatin condensed cell percentages (Fig. 9).

## Discussion

Evasion of cell death has been described as a leading hallmark of cancer [4]. Cancer cells typically modulate cell death or apoptosis pathways transcriptionally, translationally, and post-translationally to escape cell death by increasing or decreasing expression of anti-apoptotic or pro-apoptotic genes, respectively. Moreover, cancer cells may stabilize or de-stabilize anti-apoptotic or pro-apoptotic proteins, respectively [59]. As a result, the dysregulation of apoptosis would lead to continued cell proliferation and enhanced cancer development. Consequently, whichever treatment strategies that can recover the apoptotic signaling pathways may be a good advantage for cancer management. Many anti-cancer reagents included chemotherapeutic and targeted therapeutic drugs have their mechanism based on apoptosis induction effects. Chemotherapy has functions to induce cancer cell apoptosis through various mechanisms whereas targeted therapy targets on various molecules in essential pathways such as survival pathways and apoptotic pathway [60].

Aside from apoptosis, autophagy has been introduced as an important mechanism which relates to cell death. Autophagy normally is the pro-survival process by removal of potentially harmful and damaged cellular components. However, the role of autophagy in survival and death is dependent on its extent, duration, and the cellular context. Several evidences suggest that over-activation of the autophagic pathway with long duration and high intensity of cellular context would lead to cell death [61]. Similar threshold effects on cell survival and cell death are observed in stress responses such as the endoplasmatic reticulum (ER) stress response and activation of p53 [62]. In some scenarios, autophagy is crucial for the activation of other cell death pathways rather than being a direct cause of cell death. Several studies reported that autophagy and apoptosis occurs in the collaborative way during the treatment of anticancer compound [9]. The term ‘autophagy-associated cell death’ is defined as the induction of autophagy that co-occurs with the induction of apoptosis but does not play active role in it [10]. Given the function of autophagy, the compound that activate autophagy-associated cell death mechanism might carry potential relevance for cancer therapy.

Napabucasin (NB) is a plant-produced furanonaphthoquinone found in Bignoniaceae family and several botanical sources [25–31]. NB is introduced as a small molecule inhibitor of signal transducer and transcription factor 3 (STAT3), a key regulator of stemness, which activities has been shown to induce apoptosis and impair self-renewal in cancer stem cells (CSCs) of several types of cancers [32–35]. Recent study has reported that NB could inhibit metastasis in mouse model of colon cancer and also decrease CSCs related molecules such as Nanog, sex-determining region Y-box protein 2 (Sox2), c-Myc, and β-catenin [35]. NB also downregulated MUC1 which sensitized the stemness-high cancer cells to paclitaxel and inhibited spherogenesis [63]. Moreover, NB elicited inhibition effects of stemness gene expression, depletion of CSCs, and overcoming cisplatin resistance in NSCLC [33, 36, 37]. Recently, phase I-III clinical trials have been reported an efficiency of NB as both monotherapy and in combination with standard chemotherapies [64]. Although the effects of NB on CSCs have been reported in several cancer types, the other mechanisms and its structure–activity relationships (SARs) have not been elucidated yet.

In this study, we firstly confirm the cytotoxicity and apoptosis induction effects of NB. Not only NB was tested, but a newly synthesized NB derivative form noted as NB-acid with a substituted hydroxyl moiety of carboxyl group was used for comparing SARs. After treating the NSCLC cells with both compounds, NB had definitely shown a potential to induce cytotoxicity, inhibit colony formation, and induce apoptosis in NSCLC cell lines with a greater efficiency than NB-acid. It could be identified from the lower IC_50_, the lower colony number, and the much more dead cells at the same concentrations of NB-acid (Figs. 1C, 2A-B, and 3A). Additionally, an increase of cleaved PARP protein levels that proved the apoptosis induction activities of NB were shown at the 1 µM which is the lower concentration than NB-acid (10 µM). When the apoptotic related proteins were investigated, NB was found to decrease the expression levels of both anti-apoptotic proteins Mcl-1 and Bcl-2, whereas NB-acid reduced only Bcl-2 levels (Fig. 3B). These finding suggested that a structure modification from changing methyl moiety of acetyl group of NB to hydroxyl moiety of carboxyl group of NB-acid (Fig. 1B) would greatly affect the compound activities [65]. This might be related with the properties of their functional group since carboxyl group (− COOH) is classified as a polar substituent greater than that of acetyl group (–COCH_3_). As it is known that cell membranes are composed by lipids, lipid-soluble or nonpolar molecules can easily pass through the membrane because it dissolves in the hydrophobic and non-polar portion of the membrane lipid bilayer. In addition, acetyl functional group are frequently used to mask other compound to increase cell permeability. Therefore, it is not surprising that NB would have more influence on NSCLC cells. This study was accorded with the SARs theory, which is essential for new drug discovery and provides beneficial information for further anti-cancer drug modification and drug development. Consequently, only NB was chosen to be further investigated.

Autophagy induction by NB was next clarified as an increase of the autophagic vacuoles in NSCLC cells after staining with monodansylcadaverine (MDC) (Fig. 4A). The conversion of LC3B I to LC3B II in Western blot analysis confirmed the autophagy mechanism (Fig. 4B). To be concluded, NB had a potency to induce both apoptosis and autophagy. In general, apoptosis and autophagy are the key processes to regulate cell fate. Although the relationship between this two systems has not cleared yet, their processes is somehow connected with the same up-stream regulators known as PI3K/Akt/mTOR signaling pathway. This survival signaling cascade is usually found overactivated among several cancer types including NSCLC [17]. In PI3K/Akt pathway, growth factor receptor protein tyrosine kinases are activated, resulted in autophosphorylation on tyrosine residues. PI3K is recruited by directly binding to phosphotyrosine consensus residues of growth factor receptors. This leads to allosteric activation of the catalytic subunit. The second messenger phosphatidylinositol-3, 4, 5-trisphosphate (PIP3) is produced. The lipid products of PI3K and PIP3 recruit a subset of signaling proteins with pleckstrin homology (PH) domains to the membrane, including Akt [66]. Evidences suggested that increase of Akt activation could up-regulate anti-apoptotic protein Bcl-2 expression levels and down-regulate pro-apoptotic protein Bax [18]. mTOR is usually assembled with other proteins into two main complexes; mTOR complex 1 (mTORC1) and mTOR complex 2 (mTORC2). Akt activation also leads to stimulate mTORC1 activity by phosphorylation of tuberous sclerosis complex 2 (TSC2) and PRAS40, the negative regulators of mTOR. An increase of mTORC1 activation translationally up-regulates anti-apoptotic protein Mcl-1 expression levels [67, 68]. Sustaining activation of this pathway results in an evasion of apoptosis. For the autophagy aspect, mTORC1 is the key regulator of autophagic mechanism which is activated by Akt [20]. The mTORC1 attenuates autophagy by directly inhibition of the early steps of the process (Atg13 and ULK1 complex), and the control of the lysosomal degradation by blocking the activity of TFEB family members [56]. Unlike mTORC1, the mTORC2 is the protein complex that stabilize and activate Akt at Thr-450 and Ser-473 respectively. The lack mTORC2 phosphorylation on Akt caused Akt inactivation and might be degradation [58]. Gathering from these facts, PI3K, Akt, mTORC1, and mTORC2 are represented as attractive targets for cancer therapy involving apoptosis and autophagy. From this concept, computational molecular docking was performed to screen whether NB has an ability to bind and inhibit these proteins in the essential binding sites or not compared to the specific reference compounds. The result showed that NB did not significantly bind to PI3K when compared with wortmannin, a well-known PI3K inhibitor due to the lower binding affinity and the lack of hydrogen bond interaction (Fig. 5). However, NB was found to stably bind to Akt with the comparable affinities to reference compounds and hydrogen bond creativity at the protein active site and PH-domain (Fig. 6). Moreover, NB could also bind to both mTORC1 and mTORC2 at the catalytic subunits with hydrogen bond interaction and had higher binding affinities than ATP (Fig. 7). Thus, NB might interrupt the interaction between ATP complex and the catalytic site of mTOR in both form. Consequently, these docking results were confirmed by Western blot analysis. In the same way, NB could decrease the activation of Akt and mTOR due to the reduction of phosphorylated form and total form of these protein expression levels, whereas p-PI3K and PI3K levels had no change (Fig. 8). Hence, these results might assure that NB could bind with Akt, mTORC1, and mTORC2 and inhibit their activation. As a results, it was no surprising that the survival downstream targets of Akt signaling such as Bcl-2 and Mcl-1 were decreased at 24 h after treatment.

Akt and mTOR are the key proteins driven apoptosis and autophagy. Having established that NB could induce autophagy indicated by LC3I to LC3II conversion (Fig. 4), addition of autophagic inhibitor (chloroquine) could not rescue cell death caused by NB, suggesting that the mechanism of cell death caused by NB involves autophagy-associated death, the cell death caused by autophagy induction accompanying by apoptosis [10]. Having shown that mTOR is a potential protein target of NB, we further found that addition of known mTOR inhibitor rapamycin enhanced cell death caused by NB (Fig. 9). Rapamycin targets allosteric site of mTOR [69], while NB targets mTOR at catalytic site. It is possible that the synergistic cytotoxic effects of rapamycin and NB may cause by the different site of interaction to mTOR molecule. As mTOR is a protein functioning in cell survival, addition of mTOR inhibitor to NB-treated cell might enhance the cytotoxic effect of NB via inhibition of survival protein mTOR.

As it was suggesting that NB could cause autophagy, the diminishment of several proteins such as PARP, Akt, mTOR, and c-Myc at 24 h of 5–10 µM NB treatment (Figs. 3B and 8A) could be explained. It could be a result of autophagy and lysosomal degradation. Autophagy is known as an intracellular pathway for protein degradation via lysosomal degradation. It is well-known that autophagy is highly regulated, and the autophagy-associated protein degradation is selective [70]. The selected proteins for autophagy degradation have been shown to be at least in part regulated by the specific receptors such as p62 and other sequestosome-like receptors (SLRs) [71, 72]. The p62 interacts with LC3 at LIR motif and brings proteins to the autophagosome and therefore degradation [73, 74]. During autophagy, PARP was demonstrated to be targeted to lysosomal degradation. H_2_O_2_ was shown to trigger the nuclear translocation of PARP, and the degradation of PARP via the Chaperone-mediated autophagy pathway [75]. Therefore, it is possible that PARP and its cleaved form were diminished because of autophagy degradation. In addition, Akt was shown to accumulate in the autophagosome via the Akt–Phafin2 interaction [76]. Likewise, it was found that mTOR degradation involves lysosomal pathway [77]. c-Myc, a survival protein functioned in regulating cell proliferation and metabolism was shown to be sensitive to autophagy related protein degradation via the Chaperone-mediated autophagy (CMA) [78]. Together with these information, it is possible that the proteins absenced at 24 h in response to NB treatment (Figs. 3B and 8A) might cause by selective autophagy-dependent protein degradation.

## Conclusion

**Fig. 10:**
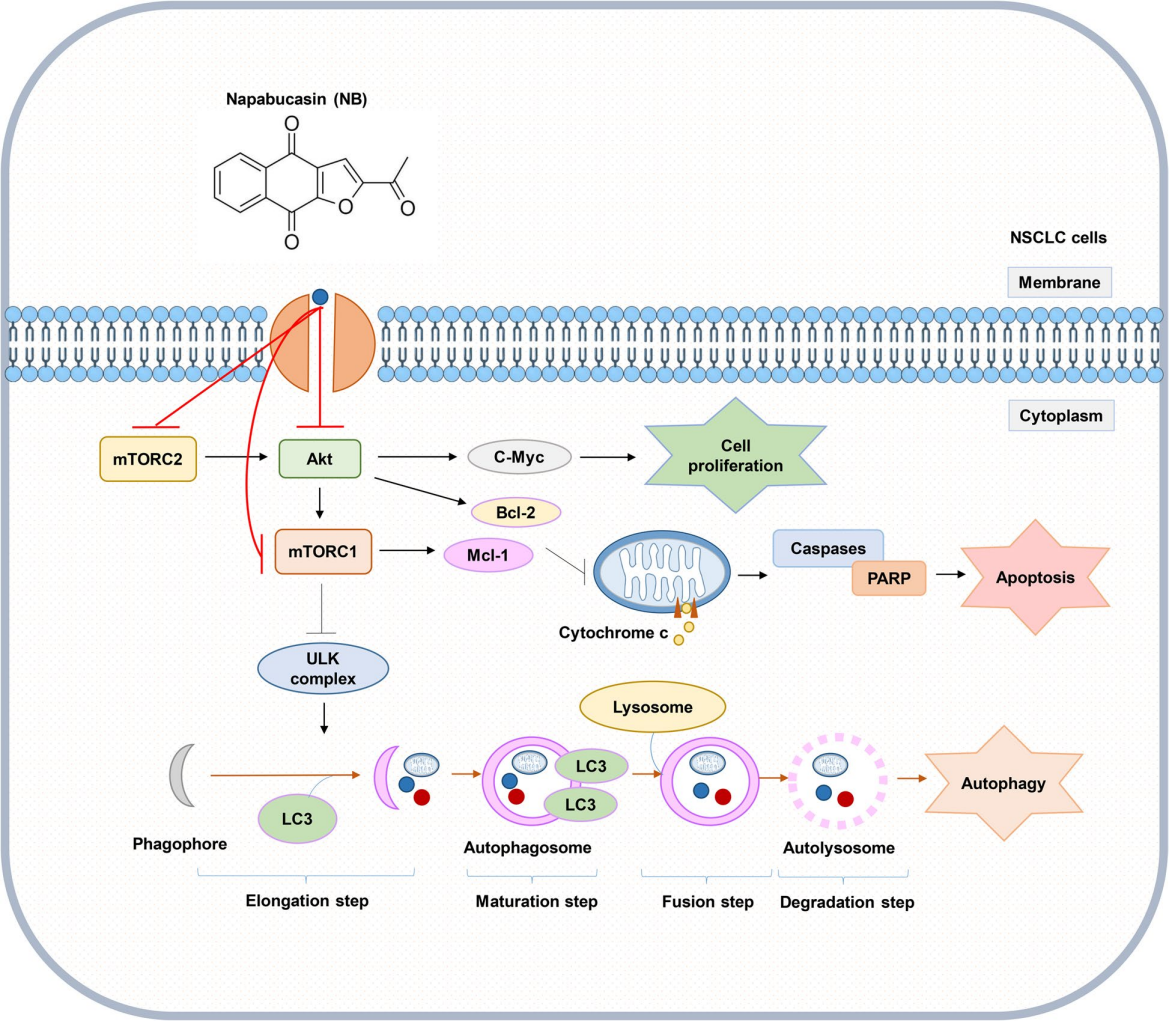
Schematic display of NB inducing autophagy and apoptosis through an inactivation of Akt and mTOR (complex 1 and 2)

In conclusion, this study presented the novel mechanism of NB for targeting Akt and mTOR which are the key regulators of apoptosis and autophagy processes. Blocking of Akt and mTOR leads to apoptosis induction and proliferative inhibition due to the lowering of anti-apoptotic proteins Bcl-2, Mcl-1, and c-Myc and leads to autophagy induction indicated by the LC3B I and LC3B II conversion. These data might be useful for emphasizing NB as a lead compound with supportive information to be further developed for targeted anti-cancer approaches (Fig. 10). Besides, the methyl moiety of acetyl group of NB was found to be necessary for the potent anti-cancer activities. These data provides beneficial information for further NB compound modification and drug development.

## Supplementary Information

**Additional file 1.**

**Additional file 2.**

**Additional file 3.**

**Additional file 4.**

## Data Availability

The datasets used and/or analysed during the current study are available from the corresponding author on reasonable request.
