# Supplementary figures and images for: Novel mechanism of napabucasin, a naturally derived furanonaphthoquinone: apoptosis and autophagy induction in lung cancer cells through direct targeting on Akt/mTOR proteins
Source: BMC Complement Med Ther. 2022 Sep 30;22:250. doi: 10.1186/s12906-022-03727-6 (PMC9524025; doi:10.1186/s12906-022-03727-6)

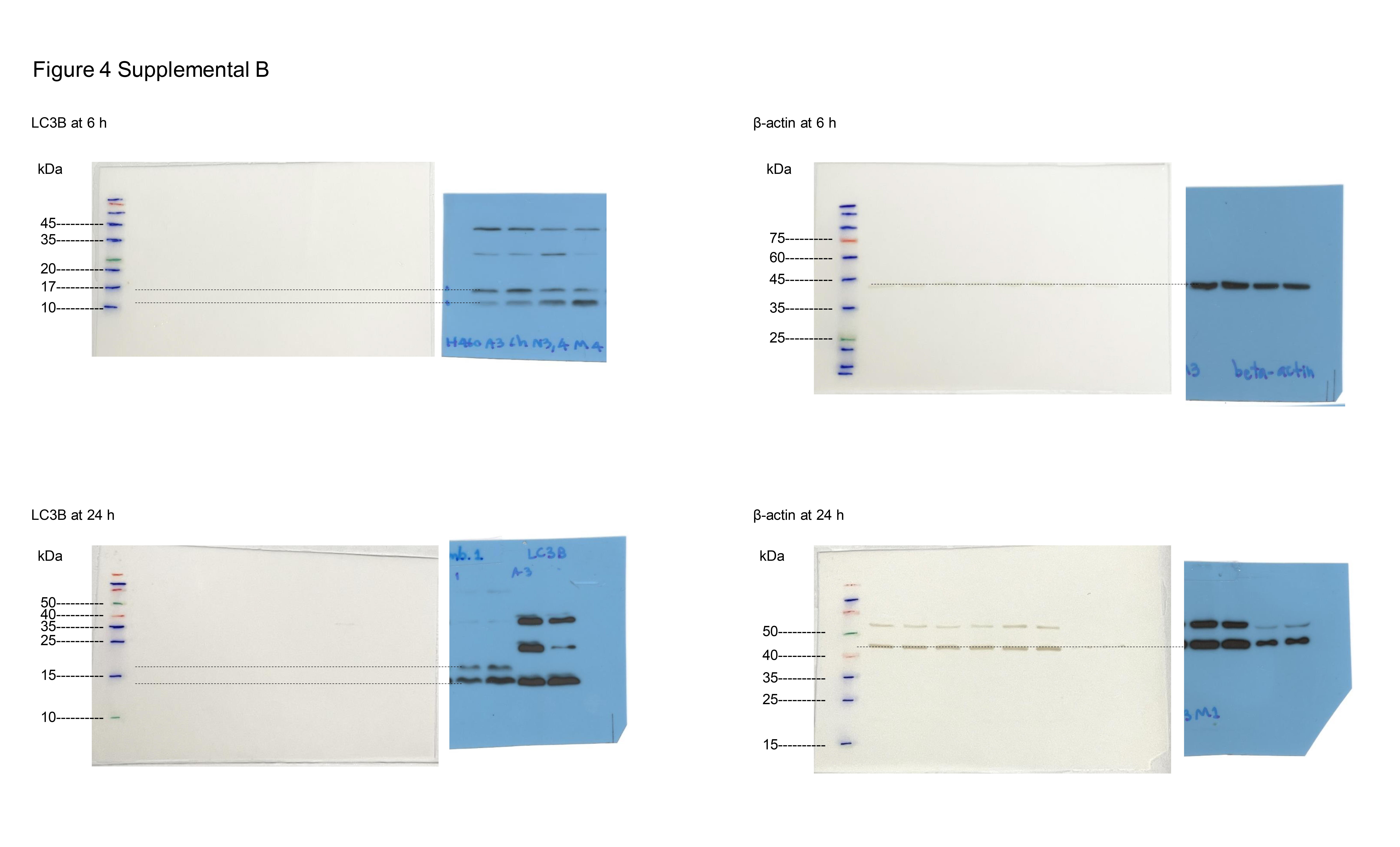

Supplement: Supplementary file 2 — Additional file 2. [file 12906_2022_3727_MOESM2_ESM.jpg]

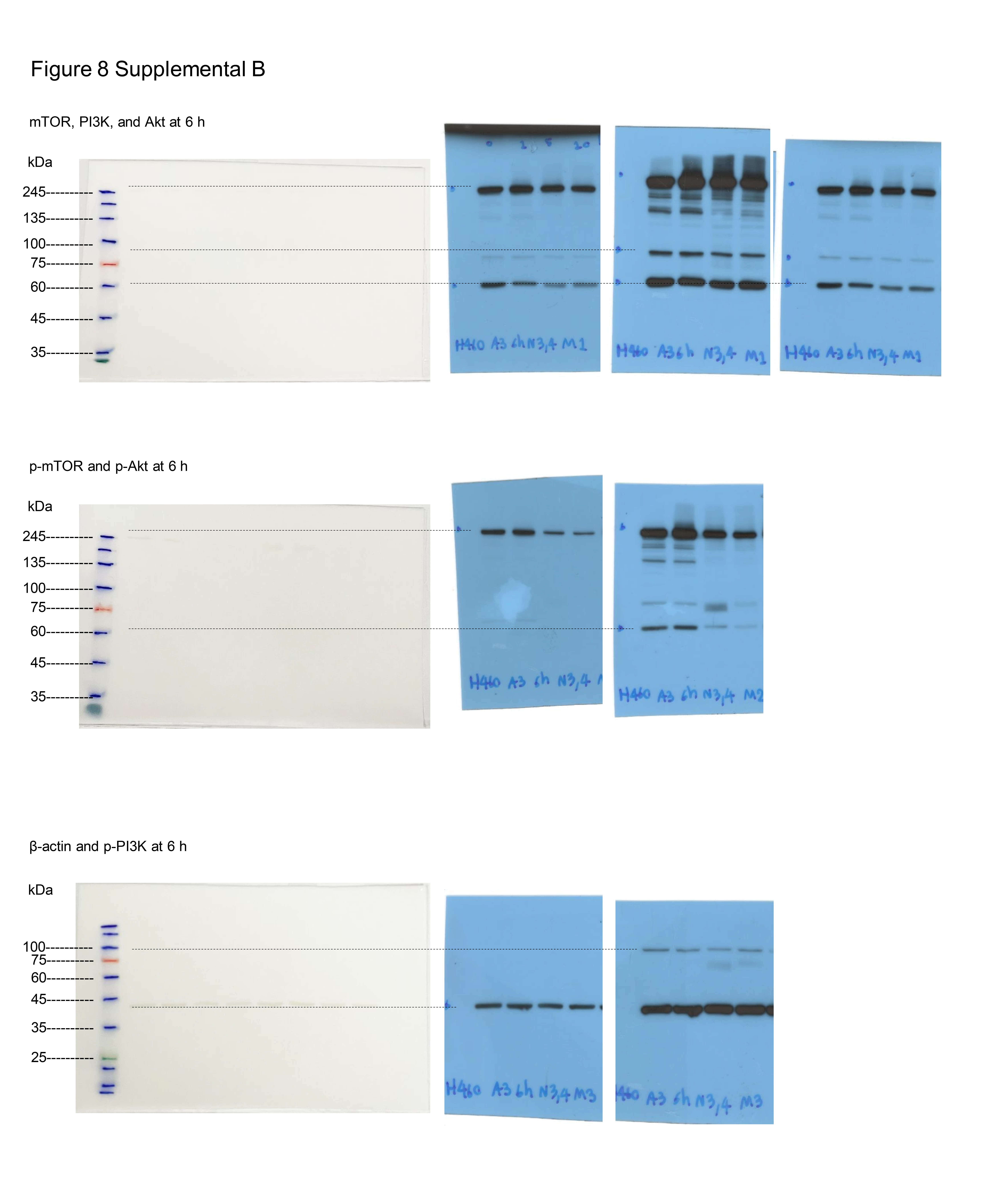

Supplement: Supplementary file 4 — Additional file 4. [file 12906_2022_3727_MOESM4_ESM.jpg]
